# Guidance for Assessment of Poliovirus Vaccination Status and Vaccination of Children Who Have Received Poliovirus Vaccine Outside the United States

**DOI:** 10.15585/mmwr.mm6601a6

**Published:** 2017-01-13

**Authors:** Mona Marin, Manisha Patel, Steve Oberste, Mark A. Pallansch

**Affiliations:** 1 Division of Viral Diseases, National Center for Immunization and Respiratory Diseases, CDC.

In 1988, the World Health Assembly resolved to eradicate poliomyelitis (polio). Since then, wild poliovirus (WPV) cases have declined by >99.9%, from an estimated 350,000 cases of polio each year to 74 cases in two countries in 2015 (*1*). This decrease was achieved primarily through the use of trivalent oral poliovirus vaccine (tOPV), which contains types 1, 2, and 3 live, attenuated polioviruses. Since 2000, the United States has exclusively used inactivated polio vaccine (IPV), which contains all three poliovirus types (*2**,**3*). In 2013, the World Health Organization (WHO) set a target of a polio-free world by 2018 (*4*). Of the three WPV types, type 2 was declared eradicated in September 2015. To remove the risk for infection with circulating type 2 vaccine-derived polioviruses (cVDPV), which can lead to paralysis similar to that caused by WPV, all OPV-using countries simultaneously switched in April 2016 from tOPV to bivalent OPV (bOPV), which contains only types 1 and 3 polioviruses (*5*). This report summarizes current Advisory Committee on Immunization Practices (ACIP) recommendations for poliovirus vaccination and provides CDC guidance, in the context of the switch from tOPV to bOPV, regarding assessment of vaccination status and vaccination of children who might have received poliovirus vaccine outside the United States, to ensure that children living in the United States (including immigrants and refugees) are protected against all three poliovirus types. This guidance is not new policy and does not change the recommendations of ACIP for poliovirus vaccination in the United States. Children living in the United States who might have received poliovirus vaccination outside the United States should meet ACIP recommendations for poliovirus vaccination, which require protection against all three poliovirus types by age-appropriate vaccination with IPV or tOPV. In the absence of vaccination records indicating receipt of these vaccines, only vaccination or revaccination in accordance with the age-appropriate U.S. IPV schedule is recommended. Serology to assess immunity for children with no or questionable documentation of poliovirus vaccination will no longer be an available option and therefore is no longer recommended, because of increasingly limited availability of antibody testing against type 2 poliovirus.

The widespread use of OPV, most commonly tOPV, has been critical for polio eradication efforts. However, OPV use, particularly in areas with low vaccination coverage, is associated with a low risk for reemergence of cVDPVs, which can lead to outbreaks of poliomyelitis similar to those caused by WPV (*6*). Type 2 cVDPVs in particular have accounted for >94% of all cVDPVs and have caused more than 650 polio cases since 2006, including several outbreaks in 2015 (*7*). Furthermore, type 2 cVPDVs have been detected in environmental (sewage) samples in recent years (in 2015 in Pakistan and in 2015 and 2016 in Nigeria) (*7*,*8*). To remove the risk for infection with type 2 cVDPVs, all OPV-using countries simultaneously switched from tOPV to bOPV in April 2016 (*5*). To further reduce the risk for reintroduction of type 2 polioviruses, laboratory containment activities limiting the handling of potentially infectious materials to certified poliovirus-essential facilities were initiated in 2015 (*9*). Although circulation of indigenous WPV in the United States ceased decades ago, the risk for importation of either WPV types 1 or 3 as well as cVDPVs remains (*10*). The following guidance is provided to highlight recent changes in global polio eradication program strategies and to ensure adequate vaccination according to ACIP recommendations of children who might have received poliovirus vaccination outside the United States.

## Current ACIP Recommendations for Routine Poliovirus Vaccination in the United States

In the United States, all infants and children should receive 4 doses of IPV at ages 2 months, 4 months, 6 through 18 months, and at 4 through 6 years (*2**,**3*). The final dose in the series should be administered on or after the fourth birthday, regardless of the number of previous doses, and should be given ≥6 months after the previous dose. A fourth dose in the routine IPV series is not necessary if the third dose was administered at age ≥4 years and ≥6 months after the previous dose.

Vaccines administered outside the United States generally can be accepted as valid doses if the schedule (i.e., minimum age for vaccination and intervals between doses) is similar to that recommended in the United States.* Vaccination against polio is also valid for children from countries that use an accelerated schedule, with the first dose given as early as 6 weeks and the second and third doses administered at least 4 weeks after the previous doses. The minimum interval between the third and fourth doses should be 6 months. Only written, dated records are acceptable as evidence of previous vaccination. Documentation of vaccination with OPV outside the United States should specify vaccination against all three poliovirus types. If both tOPV and IPV were administered as part of a series, the total number of doses needed to complete the series is the same as that recommended for the U.S. IPV schedule. A minimum interval of 4 weeks should separate doses in the series, with the final dose administered on or after the fourth birthday and at least 6 months after the previous dose. If only tOPV was administered, and all doses were given before age 4 years, 1 dose of IPV should be given at age ≥4 years, at least 6 months after the last tOPV dose.

## Guidance for Assessment of Poliovirus Vaccination Status and for Vaccination of Children Who Might Have Been Vaccinated Outside the United States

**Children without adequate documentation of poliovirus vaccination.** Persons aged <18 years should be vaccinated or revaccinated in accordance with the age-appropriate U.S. IPV schedule.^†^ Adverse events after administration of IPV are rare (*2*). The 2011 ACIP General Recommendations on Immunization included the option to perform serologic testing for neutralizing antibodies to poliovirus types 1, 2, and 3 to assess immunity in children without adequate documentation of vaccination against polio. Persons with protective titers against all three poliovirus types did not need to receive repeat doses, but were recommended to complete the schedule as age appropriate. In the United States, availability of serologic testing for neutralizing antibodies has been limited in certain commercial and state health department laboratories. Serologic testing for antibodies against poliovirus type 2, an assay that uses live virus, is becoming increasingly unavailable as U.S. laboratories conform to WHO’s laboratory containment strategy to destroy type 2 poliovirus in their facilities; these activities were begun in late 2015. Demonstrating antibodies to poliovirus types 1 and 3 does not reliably indicate protection against poliovirus type 2, because countries might have used a combination of monovalent oral poliovirus vaccine (mOPV), bOPV, or tOPV for routine programs and immunization campaigns. In the absence of the availability of testing for antibodies to all 3 serotypes, serologic testing is no longer recommended to assess immunity.

**Children with documentation of poliovirus vaccination.** Previous poliovirus vaccination is valid if documentation indicates receipt of IPV or tOPV. Although tOPV was used for routine poliovirus vaccination in all OPV-using countries, mOPV or bOPV often were used in vaccination campaigns. Therefore, only documentation specifying receipt of tOPV constitutes proof of vaccination according to the U.S. polio vaccination recommendations. If such documentation cannot be validated, persons aged <18 years should be revaccinated with IPV according to the U.S. IPV schedule. Consistent with the polio eradication strategy, doses of OPV administered after April 2016 would either be bOPV (used in routine immunization and campaigns), or mOPV (used in a type-specific outbreak response).

ACIP and CDC provide public health recommendations based on the best available epidemiologic and scientific data. The global switch from tOPV to bOPV will markedly reduce the risk for type 2 cVDPV reemergence and possible importation into the United States. However, until this risk is estimated by WHO to approach zero, public health authorities in the United States should continue to follow ACIP recommendations regarding poliovirus vaccination to ensure that all children living in the United States are protected against all three poliovirus types (*2**,**3*).

## Footnotes

* https://www.cdc.gov/mmwr/preview/mmwrhtml/rr6002a1.htm.

† https://www.cdc.gov/vaccines/schedules/downloads/child/0-18yrs-child-combined-schedule.pdf.
