# *Errata:* Vol. 66, No. 1

**DOI:** 10.15585/mmwr.mm6606a7

**Published:** 2017-02-17

In the report “Guidance for Assessment of Poliovirus Vaccination Status and Vaccination of Children Who Have Received Poliovirus Vaccine Outside the United States,” on page 24, under the section “**Children with documentation of poliovirus vaccination.**” the first paragraph should have read as follows:

**Previous poliovirus vaccination is valid if documentation indicates receipt of IPV or tOPV. tOPV was used for routine poliovirus vaccination before April 1, 2016 in all OPV-using countries. Therefore, if a child has documentation of receipt of an OPV dose (rather than “tOPV”) before April 1, 2016, this represents a tOPV dose and should be counted towards the U.S. vaccination schedule, unless specifically notated that it was administered during a vaccination campaign.** * **Consistent with the polio eradication strategy, doses of OPV administered on or after April 1, 2016 are either bOPV (used in routine vaccination and campaigns), or mOPV (used in a type-specific outbreak response); these doses do not count towards the U.S. vaccination requirements for protection against all three poliovirus types. Persons aged <18 years with doses of OPV that do not count towards the U.S. vaccination requirements should receive IPV to complete the schedule according to the U.S. IPV schedule.**

## Footnotes

*
**mOPV or bOPV were often used in vaccination campaigns but doses administered during vaccination campaigns are not typically recorded in parent-held records. These doses do not count towards the U.S. vaccination requirements.**

